# Metabolism Characteristics of Lactic Acid Bacteria and the Expanding Applications in Food Industry

**DOI:** 10.3389/fbioe.2021.612285

**Published:** 2021-05-12

**Authors:** Yaqi Wang, Jiangtao Wu, Mengxin Lv, Zhen Shao, Meluleki Hungwe, Jinju Wang, Xiaojia Bai, Jingli Xie, Yanping Wang, Weitao Geng

**Affiliations:** ^1^State Key Laboratory of Food Nutrition and Safety, College of Food Science and Engineering, Tianjin University of Science and Technology, Tianjin, China; ^2^State Key Laboratory of Bioreactor Engineering, East China University of Science and Technology, Shanghai, China

**Keywords:** lactic acid bacteria, degradation, products, metabolism characteristics, expanding applications

## Abstract

Lactic acid bacteria are a kind of microorganisms that can ferment carbohydrates to produce lactic acid, and are currently widely used in the fermented food industry. In recent years, with the excellent role of lactic acid bacteria in the food industry and probiotic functions, their microbial metabolic characteristics have also attracted more attention. Lactic acid bacteria can decompose macromolecular substances in food, including degradation of indigestible polysaccharides and transformation of undesirable flavor substances. Meanwhile, they can also produce a variety of products including short-chain fatty acids, amines, bacteriocins, vitamins and exopolysaccharides during metabolism. Based on the above-mentioned metabolic characteristics, lactic acid bacteria have shown a variety of expanded applications in the food industry. On the one hand, they are used to improve the flavor of fermented foods, increase the nutrition of foods, reduce harmful substances, increase shelf life, and so on. On the other hand, they can be used as probiotics to promote health in the body. This article reviews and prospects the important metabolites in the expanded application of lactic acid bacteria from the perspective of bioengineering and biotechnology.

## Introduction

In recent years, more and more attention has been paid to the metabolism of lactic acid bacteria. Lactic acid bacteria (LAB) are a type of gram-positive bacteria that use carbohydrates as the only or main carbon source (George et al., 2018). Lactic acid bacteria are generally cocci or rods, and have strong tolerance to low pH. Although lactic acid bacteria include more than 60 genera, the frequently genera occur in food fermentation generally include *Lactobacillus*, *Lactococcus*, *Leuconostoc*, *Pediococcus*, *Streptococcus*, *Enterococcus*, *Weissella*, etc. (Mokoena, 2017). But it has recently been proposed to merge *Lactobacillaceae* and *Leuconostocaceae* in one family *Lactobacillaceae*. The genus *Lactobacillus* was also reclassified into 25 genera (Zheng et al., 2020). For the taxonomy of the genus *Lactobacillus* was recently revised, the current nomenclature are used throughout this review.

As a fermentation strain, lactic acid bacteria should have several important metabolism characteristics, such as the ability to produce acid and aroma, the ability to hydrolyze protein, the ability to produce viscous exopolysaccharides and the ability to inhibit bacteria. In this review, the metabolic characteristics of lactic acid bacteria and its application in food industry were reviewed from the aspects of degradation (Table 1) and biosynthesis (Table 2) metabolism of lactic acid bacteria. We hope to summarize the new development trends and promote the contribution of lactic acid bacteria related metabolic engineering and food biotechnology to the food industry.

**TABLE 1:** The degradation of macromolecular substances in food by lactic acid bacteria.

Substance	Metabolic Engineering works	Expanding applications in the food industry	Lactic acid bacteria strains (References)
Polysaccharides	Hydrolyze polysaccharides with α-(1→4) glycosidic bonds	Hydrolyze starch or fructan in sourdough	*Weissella* (Falasconi et al., 2020) *Lactiplantibacillus plantarum* (Oguntoyinbo and Narbad, 2012)
Proteins and related Amino acids	Heterologous expression of *prtB* gene encoding the protease	Effectively hydrolyze protein in milk	*Enterococcus faecium* (Biscola et al., 2018; Kordesedehi et al., 2018; Worsztynowicz et al., 2019)
	Different bioactive peptides and the bioactivity diversity can be increased by editing the proteolytic system of *Lactococcus lactis*	Improve the *in vitro* digestibility of protein of cereal products	*Lactiplantibacillus plantarum* VTT E-133328 (Coda et al., 2015)
Other non-nutritive and harmful substances		Produce urethanase-promoted EC degradation in alcohol fermentation	*Oenococcus oeni*, *Levilactobacillus brevis*, and *Lactiplantibacillus plantarum* (Fang et al., 2019)
		Decompose phytic acid in the fermentation process of yam-based foods	*Leuconostoc lactis* CCMA 0415, *Lactiplantibacillus plantarum* CCMA 0744 (Batista et al., 2019)
		Hydrolyze bitter peptides in cheese production	*Lactobacillus helveticus* (Komprda et al., 2008)

**TABLE 2:** Substances synthesized in food by lactic acid bacteria.

Substance	Metabolic Engineering works	Expanding applications in the food industry	Lactic acid bacteria strains (References)
Lactic acid	Heterologous expression of gene encoding short-chain dehydrogenase for higher yield of D-lactic acid	Use dairy industry waste as a substrate to reduce costs	*Pediococcus acidilactici* (Qiu et al., 2020), *Lacticaseibacillus rhamnosus* B103 (Bernardo et al., 2016) *Lacticaseibacillus casei*, *Lactiplantibacillus pentosus* and *Lactobacillus sp.* (Shirai et al., 2001) *Enterococcus faecalis* (Deibel and Niven, 1964)
	Improve the yield of lactic acid by adding different nutrients such as the substrate glucose or vitamin B compounds or adopting pH control strategies	Fermentation strategies and metabolic engineering are often used to improve the yield and purity of lactic acid	*Lacticaseibacillus rhamnosus* HN001 (Wang et al., 2019), *Pediococcus acidilactici* ZY271 (Han et al., 2019) *Lactiplantibacillus pentosus* CECT4023T (Cubas-Cano et al., 2019)
Other organic acids	The organic acid (formic acid, acetic acid, propionic acid, butyric acid, and succinic acid) production of lactic acid bacteria in fish infusion broth	Detection of organic acids produced by lactic acid bacteria and improvement of food quality and safety	*Lactobacillus lactis subsp. Lactis* (Sezen et al., 2016)
	3-Hydroxypropionic acid produced through glycerol metabolism	3-Hydroxypropionic acid is an important platform chemical	*Limosilactobacillus reuteri* (Kumar et al., 2013)
	The production of lactic acid, propionic acid was and succinic acid in fermented silages	The production of organic acids in fermented fish silages replaces the need of the addition of chemical additives for acidification	*Levilactobacillus brevis, Lactiplantibacillus plantarum, Pediococcus acidilactici*, and *Streptococcus spp.*
	Heterologous expression of mvaES gene of *Enterococcus faecalis*	Synthesize mevalonate	*Enterococcus faecalis* (Wada et al., 2017)
Bacteriocin		Inhibit the growth of *Listeria monocytogenes* in raw minced beef and gilthead sea bream	*Lactiplantibacillus plantarum* TN8 (Trabelsi et al., 2019), *Latilactobacillus sakei* CTC494 (Costa et al., 2019)
	Gasserins has antibacterial activity against *Listeria monocytogenes* or *Bacillus cereus*	Gassericin A can be an important tool for food preservation	*Lactobacillus gasseri* (Pandey et al., 2013)
	Sakacin P has antibacterial activity against *Listeria monocytogenes* or *Bacillus cereus*	Sakacin P exerts its antibacterial effect in fermented sausage	*Latilactobacillus sakei* (Chen et al., 2012)
Vitamins	Add passion fruit by-product and oligofructose to soy milk can produce folic acid	Synthesize folic acid in dairy products	*Streptococcus*, *Lactobacillus* and *Lactococcus* (Khalili et al., 2020), *Lactococcus lactis* NZ9000 (Wegkamp et al., 2007),
	Insert a 1059-bp DNA fragment into the upstream regulatory region of the rib operon of *Lactiplantibacillus plantarum*	Induce the overexpression of riboflavin biosynthesis	*Lactiplantibacillus plantarum* (Ge et al., 2020)
	Purine biosynthesis can trigger riboflavin secretion more effectively in *Lactococcus lactis*		*Lactococcus lactis* JC017 (Chen et al., 2017)
Extracellular polysaccharides	Synthesize glucan using sucrose	Synthesize isomalto-/malto-polysaccharides by using different substrate	*Leuconostoc mesenteroides* (Yan et al., 2018)*Lactobacillus crispatus* (Hidalgo-Cantabrana et al., 2019)*Limosilactobacillus reuteri* 35-5 (Bai et al., 2016)
		Increase the extracellular polysaccharide content of yogurt	*Streptococcus thermophilus* zlw TM11 and *Lactobacillus delbrueckii subsp. bulgaricus* 34.5 (Han et al., 2016)
		Has strong inhibitory activity with a variety of pathogenic bacteria	*Lactococcus lactis* F-mou (Nehal et al., 2019), *Lactiplantibacillus plantarum* BR2 (Sasikumar et al., 2017)
	Two glycosyltransferases participate in the formation of glucan	Exploration of a new way of glucan biosynthesis	*Lactobacillus johnsonii* (Mayer et al., 2020)
	Glucan will extend to the crumb porosity of bread	Improvement of bread texture	*Limosilactobacillus reuteri* (Leemhuis et al., 2014)
γ-aminobutyric acid	Mutations in the GadA or *gadR* gene facilitate the conversion of L-monosodium glutamate (MSG) to GABA	Increase the GABA content in fermented cereals	*Levilactobacillus brevis* (Lyu et al., 2019) *Levilactobacillus brevis* D17 (Gong et al., 2019)
	GadC transports L-glutamate into the cell		*Lactococcus lactis* (Small and Waterman, 1998)
	Glutamate decarboxylase and pyridoxal-5′-phosphate participate in the decarboxylation reaction of L-glutamate		*Lactococcus lactis* (Cui et al., 2020)
	The cell immobilization technology increase GABA production		*Levilactobacillus brevis* RK03 (Hsueh et al., 2017) *Levilactobacillus brevis* (Shi et al., 2017)
Flavor substances	SHMT gene encodes a serine hydroxymethyltransferase with threonine aldolase activity	Produce flavor substances (2,3-butanedione and 2,3-pentanedione, etc.) in wine, vinegar, bread, sourdough and cheese	*Streptococcus thermophilus* (Chaves et al., 2002), (Bancalari et al., 2017)
	Heterologous expression of thl, hbd, and crt which encode thiolase, β-hydroxybutyryl-CoA dehydrogenase, and crotonase, and the Treponema denticola for higher yield of N-butanol		*Levilactobacillus brevis* (Li et al., 2020),*Lacticaseibacillus casei*, *Lacticaseibacillus rhamnosus* and *Streptococcus thermophilus* (Bancalari et al., 2017),*Streptococcus thermophilus* and *Lacticaseibacillus casei* (Chammas et al., 2006)
Antioxidant substances	*Lactiplantibacillus plantarum* fermentation significantly enhanced the ability to scavenge free radical’s DPPH when the fermenting conditions were optimized by the method of responsive surface design in fermenting sheep bone	Produce antioxidant substances (active phenol metabolites, chlorogenic acid glucoside, sulforaphane) have a variety of beneficial effects on the human body	*Lactiplantibacillus plantarum* (Ge et al., 2019; Mu et al., 2019; Ryu et al., 2019), *Lacticaseibacillus rhamnosus*, *Lactobacillus acidophilus* (Kêska and Stadnik, 2018), *Leuconostoc mesenteroides* (Nam et al., 2017)
	Metabolize phenolic acid by decarboxylase and reductase	Reduce the damage of phenolic substances to the plasma membrane and cell wall of lactic acid bacteria	*Levilactobacillus brevis*, *Limosilactobacillus fermentum* and *Lactiplantibacillus plantarum* (Filannino et al., 2018)
	Hydroxycinnamic acid (*P*-coumaric, ferulic acid and caffeic acid) can be degraded.		*Lactiplantibacillus plantarum* NC8 (Barthelmebs et al., 2000)
	Hydroxybenzoic acid (gallic acid and protocatechuic acid) can be degraded.		*Lactiplantibacillus plantarum* CECT 748T (Rodriguez et al., 2008) *Lactiplantibacillus plantarum* (Whiting and Coggins, 1971)
	Convert oxidized glutathione taken from the environment into reduced glutathione	Promotion of glutathione synthesis in industry	*Limosilactobacillus fermentum CECT* 5716 (Surya et al., 2018) *Streptococcus thermophilus* (Qiao et al., 2018)
	Mutant strain *Fructilactobacillus sanfranciscensis* DSM20451 *ΔgshR* lacking the glutathione reductase gene	Increase dough rheology; promote the hydrolysis of egg white protein; improve the acid resistance of lactic acid bacteria	*Latilactobacillus sakei* and *Fructilactobacillus sanfranciscensis* (Loponen et al., 2008) *Fructilactobacillus sanfranciscensis* (Xu et al., 2018) *Ligilactobacillus salivarius* (Lee et al., 2010)

## Degradation of Macromolecules

### Degradation of Indigestible Polysaccharides

Polysaccharides are polymers composed of more than ten identical or different monosaccharide units linked by α - or β - glycosidic bonds (Gerwig, 2019). In plants, polysaccharides include starch, cellulose, fructan, hemicellulose and so on. The degradation of polysaccharides by lactic acid bacteria depends on various hydrolases. In fermented food, the decomposition of polysaccharides can provide energy for lactic acid bacteria and provide a variety of beneficial substances for human beings. Different kinds of lactic acid bacteria can metabolize different polysaccharides, which determines the different application prospects of related strains in food industry (Velikova et al., 2016).

In fermented food processing, the degradation of polysaccharides by lactic acid bacteria can produce monosaccharides or lactic acid, etc., which can improve the quality of food. For example, the ability of lactic acid bacteria to degrade polysaccharides has be used in beverage processing to replace the use of the enzymes from molds that decompose polysaccharides. In addition, some genera of lactic acid bacteria are generally regarded as probiotics in the intestine, include *Lactobacillus*, *Enterococcus*, *S*treptococcus, Pediococcus, Leuconostoc (Fijan, 2014). The growth of the probiotics can be promoted by some kinds of polysaccharides, which be defined as prebiotics. In recent years, with the in-depth study of intestinal microbial ecology, research on commercial prebiotic oligosaccharides has increased greatly. The nature of lactic acid bacteria in degrading polysaccharides has attracted more and more attention, not only in the food and fermentation industries, but even in the medical and health-related industries.

In the past, it was thought that lactic acid bacteria made a greater contribution to fermented dairy products, and had a weak ability to hydrolyze sugars and proteins in grains. But it was found that there is a corresponding starch metabolism pathway through the analysis of the KEGG metabolic pathway of lactic acid bacteria. It is also been proved that starch can be hydrolyzed by extracellular enzymes secreted by lactic acid bacteria (Gänzle and Follador, 2012). Due to the important role played by the starch and other polysaccharides hydrolysis ability in starch-rich sourdough, this part will focus on the metabolic properties of lactic acid bacteria on starch. Starch includes amylose composed of α-(1→4) glucose chain and amylopectin composed of α-(1→4) glucose main chain and α-(1→6) glucose side chain (van der Maarel et al., 2002). According to the different types of glycosidic bonds in amylose and amylopectin, their hydrolysis require amylase (both alpha and beta type) and amylopullulanase, respectively. Lactic acid bacteria also play an important role in the fermentation of sourdough. Common bacterial species for starch hydrolysis include *Lactiplantibacillus plantarum* and *Levilactobacillus brevis* (Gänzle and Zheng, 2019). *Lactiplantibacillus plantarum* can produce amylase to hydrolyze starch into dextrin, and finally into glucose (Oguntoyinbo and Narbad, 2012). There is a potential gene encoding maltogenic amylase in the *Weissella* genome, which is used to hydrolyze starch in sourdough (Falasconi et al., 2020).

### Degradation of Proteins

**FIGURE 1:**
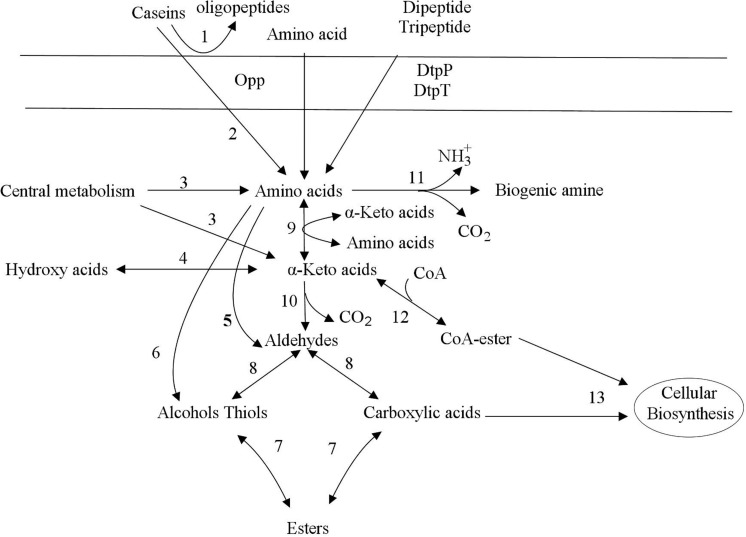
Decomposition of protein and metabolism of amino acids (Smit et al., 2005). Proteolysis in lactic acid bacteria is initiated by cell envelope proteinase (CEP), which degrades proteins into oligopeptides. The second stage of protein degradation is the transfer of dipeptides, tripeptides, and oligopeptides into cells. Three transport systems have been found in lactic acid bacteria, namely oligopeptide, dipeptide and tripeptide transport systems (Opp, DtpP, and DtpT, respectively). Finally, the Pep family hydrolyzes dipeptides, tripeptides, and oligopeptides into amino acids. The metabolism of amino acids includes deamination and decarboxylation. The deamination reaction produces various α-carboxylic acids, which are involved in various metabolisms in lactic acid bacteria cells. The amino acid decarboxylation reaction produces biogenic amines, which mainly includes the transport of amino acids into the cell, decarboxylation, and transport outside the cell after being converted into biogenic amines. Transamination of amino acids leads to the formation of alpha-keto acids. Alpha-keto acids can be converted to aldehydes by decarboxylation. Aldehydes are converted to alcohols or carboxylic acids by dehydrogenation. The direct dehydrogenation of alpha-keto acids leads to the formation of hydroxy acids. 1: cell envelope proteinase, 2: peptidases, 3: biosynthetic enzymes, 4: dehydrogenase, 5: aldolases, 6: lyases, 7: acyltransferases esterases, 8: dehydrogenase, 9: aminotransferases, 10: decarboxylase, 11: deiminases decarboxylase, 12: dehydrogenase complex, 13: biosynthetic enzymes.

During food processing, the degradation of macromolecular proteins is an important process that affects food quality, food safety and food nutrition. Dairy fermentations are only food fermentation where protein hydrolysis by lactic acid bacteria is relevant, in all others, proteases from other organisms or the substrate are much more important. Proteolysis in lactic acid bacteria can be divided into several steps, including protein degradation, peptide transport, peptide degradation and amino acid catabolism (Kunji et al., 1996; Christensen et al., 1999). Figure 1 shows the metabolic pathway of lactic acid bacteria to degrade casein in milk. Proteolysis in lactic acid bacteria is initiated by cell envelope proteinase (CEP), which degrades proteins into oligopeptides. The second stage of protein degradation is the transfer of dipeptides, tripeptides, and oligopeptides into cells. Three transport systems have been found in lactic acid bacteria, namely oligopeptide, dipeptide and tripeptide transport systems (Opp, DtpP, and DtpT, respectively) (Hagting et al., 1994; Sanz et al., 2003). Peptides are degraded in cells to amino acid by a variety of peptidases, which include endopeptidases, aminopeptidases, dipeptides, tripeptidases and proline specific peptidases (Vesanto et al., 1996).

In the food industry, the degradation of proteins by lactic acid bacteria can eliminate protein allergens in food. Especially in the fermentation process of dairy products, lactic acid bacteria can degrade casein, thereby reducing the allergenicity of dairy products (Iwamoto et al., 2019). For example, certain strains of *Enterococcus faecium* isolated from fermented milk and cheese can express metalloproteases or cell envelope proteinase (CEP) (Genay et al., 2009), etc., which can effectively hydrolyze casein in milk (Biscola et al., 2018; Kordesedehi et al., 2018; Worsztynowicz et al., 2019). Lactic acid bacteria have made significant contributions not only in dairy products, but also in other fermented foods, such as fermented fruit and vegetable products and fermented grain products. For example, some strains of lactic acid bacteria isolated from sourdough can hydrolyse some of the proteins in wheat including albumins, globulins and gliadins (Stefańska et al., 2016). A strain of *Lacticaseibacillus casei* from sourdough can metabolize all the immunotoxic 33-mer peptide (97.5 ppm) derived from α2-gliadin (Alvarez-Sieiro et al., 2016a). Another study found that some lactic acid bacteria can hydrolyze the IgE binding epitopes of the protein allergens in wheat, thereby reducing the allergenicity of wheat sourdough (Stefańska et al., 2016). Therefore, it has become a new challenge to obtain strains for removing allergens in fermented foods through natural screening or metabolic engineering methods.

Lactic acid bacteria could produce a variety of substances that are beneficial to humans when hydrolyze proteins in the surrounding environment for their own growth needs (Savijoki et al., 2006). Lactic acid bacteria can improve the digestibility of protein in food and enhance the nutritional value of food protein. In the fermentation of dairy products, the lactic acid bacteria could help the human intestinal tract to absorb the amino acids in dairy products (Meisel and Bockelmann, 1999). Fermentation of faba bean flour by a strain of *Lactiplantibacillus plantarum* VTT E-133328 can improve the *in vitro* digestibility of its protein, especially the content of essential amino acids and free amino acids (Coda et al., 2015). For the by-products of pigmented wheat varieties, hull-less barley and emmer, after lactic acid bacteria fermentation combined with xylanase treatment, the protein digestibility *in vitro* can be as high as 87%, and the product has high free radical scavenging activity and high concentration of peptides and free amino acids (Pontonio et al., 2020). Lactic acid bacteria can decompose the protein in food to produce a variety of small molecule peptides or free amino acids. For example, the main group of lactobacilli in the kefir culture has a strong decomposing effect on milk protein in milk (Dallas et al., 2016). Lactic acid bacteria such as *Lactobacillus delbrueckii*, *Lactococcus lactis*, *Lentilactobacillus kefiri*, *Streptococcus thermophilus*, *Lactobacillus acidophilus*, *Lacticaseibacillus casei*, and *Lactobacillus helveticus* can produce angiotensin converting enzyme (ACE) inhibitory peptides (Fuglsang et al., 2003; Ramchandran and Shah, 2008; Rai et al., 2017; Daliri et al., 2018; Wu et al., 2019; Rubak et al., 2020). In addition, in *Lactococcus lactis*, starter lactocepin specificity type may have an important influence on the level of bitterness in low salt-in-moisture cheeses (Pillidge et al., 2003).

### Catabolism of Amino Acids

Lactic acid bacteria can also metabolize amino acids in food, and its products include not only a variety of flavor substances, but also substances that people do not want to appear in fermented foods such as biogenic amines (BA). In lactic acid bacteria, the metabolism of amino acids includes deamination and decarboxylation (Gardini et al., 2016; Barbieri et al., 2019). The deamination reaction produces various α-carboxylic acids, which are involved in various metabolisms in lactic acid bacteria cells. Amino acids can generate a variety of biogenic amines under the action of lactic acid bacteria decarboxylase. For example, after decarboxylation, lysine, tryptophan, tyrosine, histidine and ornithine can generate cadaverine, tryptamine, tyramine, histamine, putrescine (Barbieri et al., 2019), etc., respectively, as shown in Figure 1.

Biogenic amines in food may be beneficial to the survival of lactic acid bacteria, but foods containing a large number of biogenic amines are toxic to humans (Lonvaud-Funel, 2001). The amines in fermented foods can be converted by monoamine oxidase. In cheese, *Lactobacillus* and *Enterococcus* will convert tyrosine to tyramine through the action of decarboxylase and transporter TyrP (Komprda et al., 2008). In aged cheese, *Pediococcus pentosaceus* can produce histamine in cheese (Møller et al., 2020). In view of food safety, some unfavorable metabolic activities of lactic acid bacteria under certain environmental conditions cannot be ignored. Therefore, how to balance the contribution of lactic acid bacteria to the quality of food and the accompanying potential safety issues need to be studied in the future development of lactic acid bacteria species resources and food processing (Diaz et al., 2020).

In addition, the metabolism of amino acids is of great significance for lactic acid bacteria in order to adapt to the environment (Even et al., 2002). Amino acid (especially glutamine, glutamic acid and arginine) metabolism plays an important role in the adaptation of lactic acid bacteria to the acid environment. The synthesized NH_3_ during amino acids deamination can increase the pH value inside and outside the cell, thereby protecting the cell from acid stress (Papadimitriou et al., 2016). In *Lentilactobacillus hilgardii*, the decarboxylation reaction of histidine and tyrosine also contributes to the acid resistance of the bacteria (Lamberti et al., 2011). *Levilactobacillus brevis*, *Latilactobacillus curvatus, Enterococcus faecalis*, *Lactococcus lactis* can hydrolyze agmatine into putrescine, NH_3_, CO_2_ and ATP through the AgDI pathway, which increases the pH of the cytoplasm (Papadimitriou et al., 2016). In *Streptococcus thermophilus*, the metabolism of arginine relieves the decrease of intracellular pH by consuming protons and generating NH_3_ (Huang et al., 2016).

### Conversion of Other Non-nutritive and Harmful Substances in Food

In the food and fermentation industry, lactic acid bacteria can’t only degrade the main nutritional macromolecular substances such as polysaccharides and proteins, but also can degrade some other undesirable substances. Fist of all, lactic acid bacteria can be used to inhibit the accumulation of mycotoxins during the preservation of cereal products. After artificially infected almonds with *Aspergillus flavus*, the inoculation of *Lentilactobacillus kefiri* FR7 can greatly reduce the accumulation of aflatoxin B1 and aflatoxin B2 (Ben Taheur et al., 2019). Lactic acid bacteria can also decompose harmful substances that may be produced in alcohol fermentation. For example, three lactic acid bacteria (*Oenococcus oeni*, *Levilactobacillus brevis*, and *Lactiplantibacillus plantarum*) produce urethanase-promoted EC degradation during co-cultivation with *Saccharomyces cerevisiae* to break down the potential carcinogenic ethyl carbamate (Fang et al., 2019). Lactic acid bacteria can also decompose the phytic acid that affects the taste of food and is difficult to digest. For example, in the fermentation process of yam-based foods, phytase produced by *Leuconostoc lactis* CCMA 0415, *Lactiplantibacillus plantarum* CCMA 0744 and *Limosilactobacillus fermentum* CCMA 0745 can decompose phytic acid (Batista et al., 2019). The addition of lactic acid bacteria will also reduce the possible undesirable flavors in fermented foods. For example, based on the properties of lactic acid bacteria to produce acid and aroma, the undesirable flavor caused by the fermentation of *Bacillus* can be improved through fermentation of mixed strains (Ben-Harb et al., 2019). *Lactobacillus helveticus* can be used as an auxiliary starter to hydrolyze bitter peptides in cheese production (Komprda et al., 2008). In addition, *Oxalobacter* and *Lactobacillus* species exist symbiotically in the human gut can prevent the formation of stones by producing specific enzymes that help oxalate salts degradation (Sadaf et al., 2017). Although lactic acid bacteria have shown a variety of degradation effects, because lactic acid bacteria are not traditionally considered microorganisms with strong degrading ability, the specific degradation mechanism of most harmful substances in foods still needs extensive and far-reaching research. Moreover, this degradation has limitations in efficiency, effectiveness and environmental conditions. Therefore, the use of lactic acid bacteria to achieve efficient conversion of unwanted substances in food still has certain challenges (Perczak et al., 2018).

## Products Synthesized by Lactic Acid Bacteria

### Organic Acids

In the metabolism of lactic acid bacteria, certain metabolic processes such as lactic acid fermentation can synthesize a variety of organic acids including lactic acid. Lactic acid is an important bio-based platform compound, which can be divided into D-lactic acid and L-lactic acid according to its optical rotation. It is widely used in agriculture, food, medicine, chemical industry and environmental protection. In view of the importance of lactic acid as an important industrial raw material, related synthesis research has continued to be a hot spot in recent decades. And this part will mainly discuss the application of lactic acid synthesis in the food industry and related metabolic engineering research.

**FIGURE 2:**
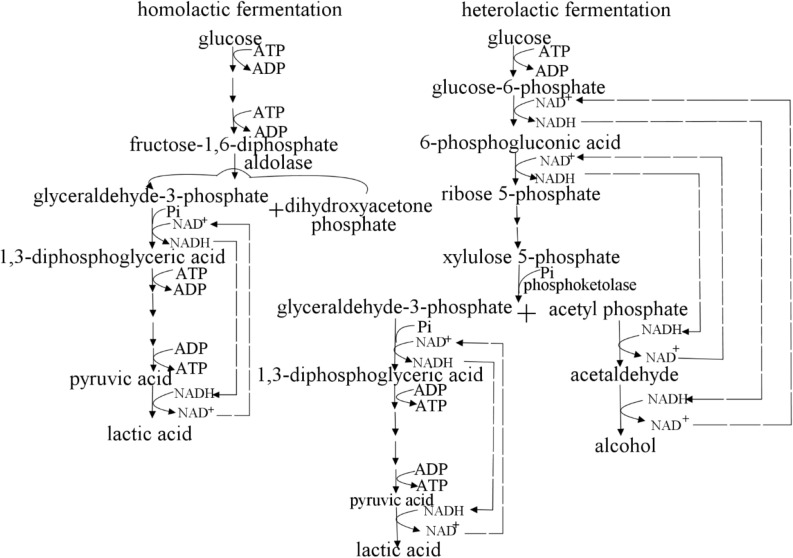
Homolactic fermentation and heterolactic fermentation. *Lactococcus spp.* performs homolactic fermentation, while *Lactobacillus* and *Leuconostoc spp.* perform heterolactic fermentation. (1) In the process of pure lactic acid fermentation, lactic acid bacteria use glucose as a carbon source to produce pyruvate through glycolysis, and then produce lactic acid under the action of lactate dehydrogenase. In theory, 1 mole of glucose produces 2 moles of lactic acid. (2) In lactic acid bacteria of the heterolactic fermentation type, glucose can be decomposed into lactic acid, ethanol, CO_2_ (in *Leuconostoc*, etc.) through the phosphoketolase (PK) pathway. In theory, 1 mole of glucose produces 1 mole of lactic acid. (3) Through the pentose phosphate (PP) pathway, glucose 6-phosphate was converted into carbon dioxide, ribulose 5-phosphate and NADPH (Eiteman and Ramalingam, 2015; Stincone et al., 2015).

According to whether aldolase is used in the process of producing lactic acid, lactic acid bacteria can be divided into homolactic fermentation and heterolactic fermentation (Figure 2). *Lactococcus* and *Lactobacillus* perform homolactic fermentation, while *Leuconostoc, Weissella* and *Oenococcus* perform heterolactic fermentation (Sridhar et al., 2005; Zaunmüller et al., 2006). (1) In the process of pure lactic acid fermentation, lactic acid bacteria use glucose as a carbon source to produce pyruvate through glycolysis, and then produce lactic acid under the action of lactate dehydrogenase (Eiteman and Ramalingam, 2015). In theory, 1 mole of glucose produces 2 moles of lactic acid. (2) In lactic acid bacteria of the heterolactic fermentation type, glucose can be decomposed into lactic acid, ethanol, CO_2_ (in *Leuconostoc*, etc.) through the phosphoketolase (PK) pathway. In theory, 1 mole of glucose produces 1 mole of lactic acid (Eiteman and Ramalingam, 2015). (3) Through the pentose phosphate (PP) pathway, glucose 6-phosphate was converted into carbon dioxide, ribulose 5-phosphate and NADPH (Eiteman and Ramalingam, 2015; Stincone et al., 2015). Lactate dehydrogenase is a key enzyme for lactic acid bacteria to transform pyruvate into lactic acid, and its stereospecificity determines the configuration of lactic acid. D-lactic acid and L-lactic acid are catalyzed by D-lactate dehydrogenase and L-lactate dehydrogenase, respectively. The optical type of lactic acid synthesized by microorganisms depends on the expression levels of D-lactate and L-lactate dehydrogenase in the strain (Kim et al., 2020).

In the food industry, there are many studies on how to improve the yield and optical purity of lactic acid during food processing (Zhang et al., 2016), and a variety of methods provide ideas from different approaches. In currently industrial production, fermentation strategies are often used to improve the yield and purity of lactic acid. These fermentation strategies include adding different nutrients such as the substrate glucose or vitamin B compounds during the fermentation process (Han et al., 2019; Wang et al., 2019), adopting pH control strategies (Cubas-Cano et al., 2019), and using dairy industry waste as a substrate to reduce costs (Bernardo et al., 2016), etc. Among them, strategies such as simultaneous saccharification fermentation (SSF) and both separate hydrolysis and fermentation (SHF) are used for the production of lactic acid to obtain products with high optical purity (>99.9%) and reduce the amount of residual sugar (Xu et al., 2016; Müller et al., 2017).

In addition to mainly producing lactic acid, lactic acid bacteria also produce acetate, propionate, 3-hydroxypropionate, formate and succinate. For example, when shrimp waste silage is used as a substrate, *Lactiplantibacillus pentosus* can also produce acetic acid (Shirai et al., 2001). *Lactobacillus* and *Limosilactobacillus reuteri* produce 3-hydroxypropionic acid through glycerol metabolism pathway (Kumar et al., 2013). Succinic acid produced by lactic acid bacteria fermentation is also one of the final fermentation products of anaerobic metabolism (Kuley et al., 2020). The organic acids usually be as a part of the flavor substances. However, due to the complexity of the types of flavor substances, we will discuss them in the following content. In general, the synthetic pathways of organic acids mainly include hetero-lactic fermentation pathways and amino acid metabolism. Firstly, the hetero-lactic fermentation will synthesize other organic acids besides lactic acid. For example, in addition to being decomposed into lactic acid, pyruvic acid can also be decomposed into acetic acid, formic acid, and ethanol under certain conditions, or decomposed into acetic acid and carbon dioxide. In addition, the α-acetolactate is formed by the conversion of excess pyruvate by α-acetolactate synthase (ALS) under aerobic conditions (Dorau et al., 2019). Secondly, some organic acids are produced in amino acid metabolism. For example, in *Lactococcus lactis*, *Lactiplantibacillus plantarum*, *Levilactobacillus brevis*, *Leuconostoc mesenteroides* and some other lactic acid strains, leucine can generate 2-ketoisocaproic acid (KICA) after transamination, and 2-ketoisocaproic acid (KICA) can be reduced to 2-hydroxyisocaproic acid (HICA). Therefore, adding the above lactic acid bacteria can increase the HICA content in fermented foods (Park et al., 2017). In *Lactiplantibacillus plantarum* LY-78, phenyllactic acid (PLA) can be accumulated as a by-product of phenylalanine catabolism (Sun et al., 2019).

Lactic acid bacteria may metabolize in the intestine to produce organic acids, which is also an important metabolic feature of probiotics. At the same time, the study of the interaction between probiotics and the intestine found that the synthesis of a variety of organic acids is affected by many environmental factors (Liao et al., 2016). Moreover, the synthesis process of a certain product is often complicated, and multiple key genes may play a synergistic role (Ali et al., 2019). Therefore, research on the synthesis of organic acids from lactic acid bacteria in the context of food or intestines, and clarifying the molecular basis of lactic acid bacteria as probiotics, are also challenging tasks in the future.

### Bacteriocin

Bacteriocins are primary metabolites of polypeptides, proteins or protein complexes synthesized by bacteria using ribosomes with antibacterial activity (Diep and Nes, 2002). Bacteriocins can inhibit the growth and reproduction of a variety of bacteria. After years of research and development, people have clear reports on the molecular composition and antibacterial mechanism of bacteriocins. The bacteriocin will interact with the cell surface to increase the permeability of the cell; inhibit the production of cell wall; inhibit the synthesis of nucleic acid; inhibit the synthesis of protein (Kumariya et al., 2019). Bacteriocins may be divided into two categories: Category I is lantibiotics containing lanthionine (such as nisin, epidermin, streptin, etc.; Deegan et al., 2006). Nisin secreted by *Lactococcus lactis* is the first lanthiococin to be identified (Mattick and Hirsch, 1947). Nisin’s mechanism of action is to cause small pores to form in the plasma membrane of gram-positive bacteria, thereby causing cell damage (Montville and Chen, 1998). The second category is bacteriocins without lanthionine (Cotter et al., 2005). And there are also a type III bacteriocins, such as helveticin M produced by *Lactobacillus crispatus* and helveticin J produced by *Lactobacillus helveticus*. The bacteriocins with biologically must be secreted out of the cell (Nes et al., 1996; Fimland et al., 2000). For example, the secretion mechanism of bacteriocins (such as class II bacteriocins) generally uses the double glycine guide sequence transport system, and some are signal-dependent, which are processed and transported by the secretory pathway transfer membrane protein (GSP) system. In addition, most of the type I and type II bacteriocins are transferred to the outside of the cell through a specific ABC transporter system (Rincé et al., 1997).

Because of its safety to the human body, some bacteriocins have been used in the food industry as bacteriostats and preservatives (Mulet-Powell et al., 1998; Cotter et al., 2005; Alvarez-Sieiro et al., 2016b). Gasserins produced by *Lactobacillus gasseri*, bacteriocin produced by *Lactococcus lactis*, and Sakacin P produced by *Latilactobacillus sakei* have antibacterial activity against *Listeria monocytogenes* or *Bacillus cereus* (Chen et al., 2012; Pandey et al., 2013; Azhar et al., 2017). *Lactiplantibacillus plantarum* TN8 can inhibit spoilage microorganisms in raw minced beef and extend the shelf life of these products (Trabelsi et al., 2019). *Latilactobacillus sakei* CTC494 can inhibit the growth of *Listeria monocytogenes* in gilthead sea bream (*Sparus aurata*), thereby improving the safety of the food (Costa et al., 2019). The bacteriocin yield of *Enterococcus mundtii* LP17 can reach 1280 AU/ml, and it has a strong inhibitory ability against *Listeria monocytogenes* (Iseppi et al., 2019). In addition to inhibiting some food contaminated strains, it has been reported that nisin may also inhibit the growth of *Latilactobacillus sakei* in ham production (Kalschne et al., 2014).

The synthesis of bacteriocins is also very important for lactic acid bacteria to perform the function of probiotics in the intestine. For example, *Levilactobacillus brevis* B50 Biocenol (CCM 8618) can significantly increase the proportion of lactic acid bacteria in the intestinal microbiota of bees, and enhance the resistance of bees to infectious diseases and harsh environments (Maruščáková et al., 2020). However, how to make lactic acid bacteria synthesize bacteriocins in a controlled dose, and efficiently and stably play the functions of antibacterial, food preservation, and intestinal health promotion in food is the future development direction of bacteriocin synthesis in the food industry.

### Vitamins

Many studies now show that lactic acid bacteria can synthesize a variety of vitamins, such as folic acid, riboflavin, vitamin C, pyridoxal (Capozzi et al., 2012), cobalamine (Taranto et al., 2003) and so on. In the food industry, the vitamins synthesized during the fermentation of lactic acid bacteria can be considered as nutritional fortification of food. This nutritional fortification expands the application of lactic acid bacteria to formulate fermented foods rich in certain vitamins for special populations (Wu et al., 2017).

Folic acid is a water-soluble vitamin B composed of three parts, purine, p-aminobenzoic acid and polyglutamic acid. As a coenzyme, it participates in the transfer of one carbon unit, thereby playing a role in the biosynthesis of nucleotides and proteins. Because humans and other mammals lack genes related to folic acid synthesis, they can only absorb folic acid in food or synthesized by intestinal flora. Most of the reported strains that can synthesize folic acid belong to the genus *Streptococcus*, *Lactobacillus*, and *Lactococcus* (Khalili et al., 2020). The folic acid synthesis pathways of lactic acid bacteria include the Pterin branches and the pABA branches, and only when these two branches function at the same time can they synthesize folic acid (Wegkamp et al., 2007). Therefore *Lactobacillus* strains can only metabolize and produce folic acid after adding p-Aminobenzoic acid (pABA) to the culture medium. Lactic acid bacteria can use a variety of substrates to synthesize or convert vitamins, such as dairy products and cereals. For example, *Streptococcus thermophilus* ST-M6 and TH-4 to add passion fruit by-product and oligofructose to soy milk can produce folic acid. Among them, passion fruit by-product as a growth factor can stimulate the synthesis of folic acid in lactic acid bacteria (Albuquerque et al., 2017).

Riboflavin can be produced by many microorganisms including fungi (such as yeast) and bacteria. In lactic acid bacteria, riboflavin synthase coding genes are clustered on a *rib* operon, and its products (RibC, RibB, RibA, and RibH) can catalyze the conversion of GTP and 5-phosphate ribose into riboflavin. In the study of constructing genetically engineered strains with high production of riboflavin, it was found that certain changes in DNA regions related to regulation will affect the synthesis of riboflavin in lactic acid bacteria. For example, by inserting a 1059-bp DNA fragment into the upstream regulatory region of the rib operon of *Lactiplantibacillus plantarum*, the amount of riboflavin produced by the mutant strain will be higher than that of the wild type (Ge et al., 2020). Another study found that in the *Lactococcus lactis* JC017 mutant, purine starvation induced the overexpression of riboflavin biosynthesis cluster *ribABGH*, indicating that mutations that inhibit purine biosynthesis can trigger riboflavin secretion more effectively (Chen et al., 2017).

In addition to folic acid and riboflavin, lactic acid bacteria can also produce other types of vitamins. For example, the aerobic fermentation of *Lactococcus lactis* subsp. *cremoris* MG1363 with fructose or trehalose as a carbon source can synthesize vitamin K2 (Liu et al., 2019). A strain of *Latilactobacillus sakei* UONUMA promotes the content of vitamin B2 (riboflavin), B3 (nicotinic acid and nicotinamide), and B6 (pyridoxine) in the traditional sweet Japanese beverage (koji amazake) (Oguro et al., 2017). *Limosilactobacillus reuteri* CRL 1098 and *Lactobacillus coryniformis* CRL 1001 are well-known cobalamin producing strains (Torres et al., 2018).

### Exopolysaccharides

Polysaccharides are macromolecular substances produced by the polymerization of multiple monosaccharides or their derivatives. In recent years, exopolysaccharides have been favored because of their excellent physical properties and probiotic functions (Salazar et al., 2016). A variety of lactic acid bacteria can synthesize different kinds of exopolysaccharides, such as *Streptococcus thermophilus*, *Limosilactobacillus reuteri, Lacticaseibacillus casei*, *Lactiplantibacillus plantarum* and so on. The exopolysaccharides of lactic acid bacteria are not only related to the adhesion of lactic acid bacteria, but also give new characteristics to fermented foods (Majee et al., 2017). Many review articles discussed the important influence of exopolysaccharides on the physiological function of lactic acid bacteria (Ruas-Madiedo and de los Reyes-Gavilán, 2005; Górska et al., 2007; Patel et al., 2012; Caggianiello et al., 2016; Rahbar Saadat et al., 2019; Zhou et al., 2019; Wu et al., 2021). In view of the potential applications of the physical, chemical and biological properties of exopolysaccharides in fermented foods, this section introduces exopolysaccharides and their synthetic strains.

**FIGURE 3:**
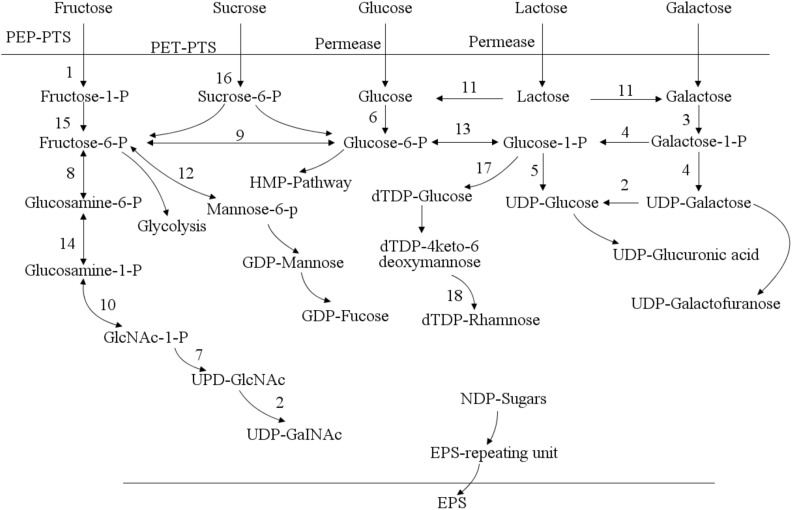
Pathways of sugar metabolism and exopolysaccharides (EPS) biosynthesis in *Streptococcus thermophilus* (Laws et al., 2001). In *Streptococcus thermophilus*, exopolysaccharides is synthesized through a series of complex intracellular enzyme interactions. It is exported to the extracellular environment in the form of macromolecules through a special lipid carrier. The biosynthetic pathway of exopolysaccharides includes sugar entry into the cytoplasm, sugar-1-phosphate synthesis, polysaccharide synthesis and exopolysaccharides export. 1: fructokinase; 2: UDP-galactose-4-epimerase; 3: galactokinase; 4: galactose-1-phosphate uridylyltransferase; 5: UDP-glucose pyrophosphorylase; 6: glucokinase; 7: N-acetylglucosamine-1-phosphate uridyltransferase; 8: glucosamine-6-phosphate deaminase; 9: glucose-6-phosphate isomerase; 10: glucosamine-1-phosphate N-acetyltransferase; 11: β-glalactosidase; 12: mannose-6 phosphate isomerase; 13: α-phosphoglucomutase; 14: phosphoglucosamine mutase; 15: phosphomutase; 16: sucrose-6-phosphate hydrolase; 17: dTDP-glucose pyrophosphorylase; 18: UDP-glucose 6-dehydrogenase.

The genome of lactic acid bacteria contains one or more clusters of exopolysaccharides gene synthesis (Cerning, 1990). A typical polysaccharide synthesis gene cluster generally includes genes related to sugar nucleotide synthesis, glycosyltransferase genes, and polysaccharide synthesis regulatory genes (Boels et al., 2001). Take the hetero-exopolysaccharide (HepS) as an example, the synthesis pathway (Figure 3) mainly includes the synthesis of precursor nucleotide sugars, the initiation and extension of repeating units, the inversion and polymerization of repeating units, and the output of polysaccharides. In the synthesis of exopolysaccharides, sugars are first transported to the cytoplasm and sugar-1-phosphate is synthesized. Phosphoenolpyruvate transport system can transfer phosphate groups to sugar during sugar transport (Boels et al., 2001). It is subsequently activated into sugar-nucleotide structural units (such as UDP-glucose, GDP-mannose, UDP-galactose, dTDP-rhamnose, etc.) and then polymerized in a certain order, and finally secreted and exported to form exopolysaccharides. In addition to the monosaccharide structural units mentioned above, the monosaccharide components of exopolysaccharide of lactic acid bacteria also include: fructose, glucuronic acid, fucose, N-acetylglucosamine, and N-acetylgalactosamine (Boels et al., 2001).

Exopolysaccharides (EPS) can be used as an additive in the food industry, especially in the fermented dairy industry. For example, glucan can be used as a gelling agent, stabilizer, thickener and emulsifier in the production of food and cosmetics. Similarly, its production strains can also be used in the processing of fermented foods. For example, some starters that can synthesize EPS (such as *Streptococcus thermophilus* zlw TM11 and *Lactobacillus delbrueckii subsp. bulgaricus* 34.5) can increase the exopolysaccharides content of yogurt and improve the syneresis, texture and sensory of yogurt (Han et al., 2016). A new type of EPS produced by a strain of *Lactococcus lactis* F-mou (LT898177.1), showing good water and oil holding capacities, high antioxidant efficiency and excellent anti-clotting activity, and has strong inhibitory activity with a variety of pathogenic bacteria (Nehal et al., 2019). The high molecular weight EPS synthesized by *Lactiplantibacillus plantarum* BR2 has strong antioxidant activity, radical scavenging activity, and no cytotoxicity (Sasikumar et al., 2017). Adding sucrose to the dough can promote the production of glucan, and the special effect of the glucan structure may extend to the pores of the crumbs, thereby improving the texture of the bread (Chen et al., 2016). In short, exopolysaccharides and their production strains have important potential applications in the food industry, but due to the complex structure of exopolysaccharides, the relationship between the synthesis mechanism and functional properties of exopolysaccharides is also the focus of future research.

### Gamma-Aminobutyric Acid

Gamma-aminobutyric acid (γ-aminobutyric acid, GABA) is a non-protein natural amino acid that is widely found in nature. GABA is an important neurotransmitter in the central nervous system, and its concentration in the human brain is related to many diseases. L-Glu decarboxylation reaction is the main manner of intracellular GABA synthesis. Glutamate decarboxylase (GAD) is the key rate-limiting enzyme that catalyzes the production of GABA. GAD (Glutamate decarboxylase) and pyridoxal-5’-phosphate participate in the decarboxylation reaction of L-glutamate to generate γ-aminobutyric acid (Small and Waterman, 1998). Several important genes that regulate GABA synthesis have been discovered. For example, in *Levilactobacillus brevis*, mutations in the GadA gene facilitate the conversion of L-monosodium glutamate (MSG) to GABA (Lyu et al., 2019). In addition, in the high GABA production *Levilactobacillus brevis* D17, the potential transcriptional regulator gene *gadR* can control the synthesis of GABA and the acid resistance of the strain, and the inactivation of *gadR* completely eliminates the synthesis of GABA (Gong et al., 2019).

At present, in the fermentation industry, *Levilactobacillus brevis* is generally used to produce GABA alone or in combination with other strains. Among them, *Levilactobacillus brevis* TCCC 13007 can convert the substrates L-glutamic acid and monosodium glutamate into GABA, and the final titer reaches 201.18 g/L, and the molar bioconversion ratio is 99.4% (Shi et al., 2017). In addition, the cell immobilization technology also plays an important role in the optimization of GABA production (Hsueh et al., 2017). In the food industry, some microorganisms with high safety that can produce GABA can be used in the production of functional health food ingredients. For example, some lactic acid bacteria can increase the GABA content in fermented cereals. Cheese, yogurt and fermented milk produced by fermentation of lactic acid bacteria can also become GABA-enriched products (Yunes et al., 2016). In traditional Chinese fermented soybean (Sufu), *Levilactobacillus brevis* and *Bacillus subtilis* can be inoculated together to increase the concentration of GABA in the product and reduce the concentration of harmful substances (histamine and serotonin) (Bao et al., 2020). In addition, in traditional Korean soybean paste, the co-fermentation of *Levilactobacillus brevis* GABA100 and *Aspergillus oryzae* KACC 40250 accelerates the conversion of monosodium glutamate and soybean isoflavone glycosides to GABA and soybean isoflavone aglycones (Li et al., 2017).

### Flavor Substances

The initial recognition of lactic acid bacteria may be due to its great contribution to humans, that is, to transform perishable milk into flavored yogurt with extended shelf life (Ibarra et al., 2012). Although yogurt has become very common in daily life, the important message brought by yogurt is that lactic acid bacteria have the potential to transform food ingredients into flavor substances. The flavor substances produced by lactic acid bacteria include organic acids, alcohols, ketones and esters (Coolbear et al., 2011). There are generally four ways of formation of flavor substances in food, biosynthesis, enzymatic action (Smit et al., 2005), oxidative decomposition and pyrolysis. In fermented foods (such as fermented dairy products, kimchi, vinegar, and fermented dough) (Azam et al., 2017), lactic acid bacteria either act as the dominant bacteria or work in concert with other dominant bacteria to produce acetaldehyde, diacetyl and other flavor substances through biosynthesis and enzymatic action. The biosynthesis of flavor substances mainly depends on two types of metabolic pathways, one is the citric acid metabolism pathway, and the other is the amino acid metabolism pathway.

#### The Metabolism of Citric Acid Produces Flavor Compounds

**FIGURE 4:**
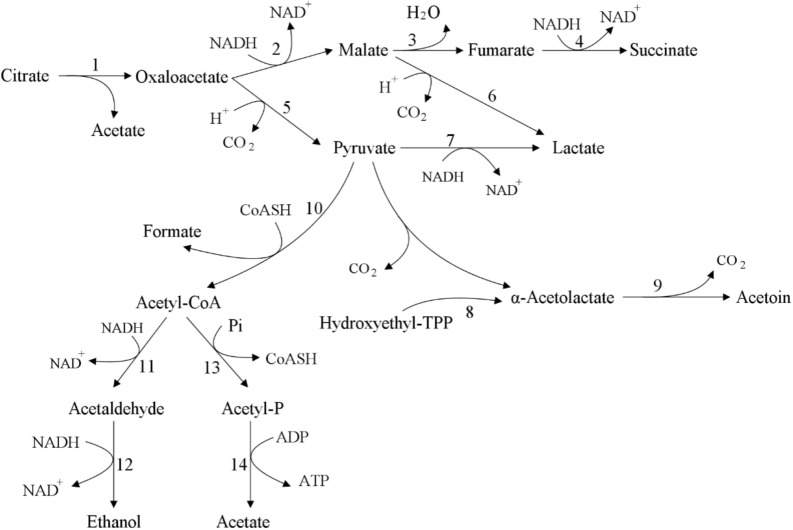
Citric acid metabolism (Gänzle, 2015). The citric acid in lactic acid bacteria is converted into succinate, lactate, acetate, and ethanol or acetylacetone through the intermediate metabolite oxaloacetate. 1: citrate lyase; 2: malate dehydrogenase; 3: fumarate hydratase; 4: succinate dehydrogenase; 5: oxaloacetate decarboxylase; 6: malolactic enzyme; 7: lactate dehydrogenase; 8: acetolactate synthase; 9: acetolactate decarboxylase; 10: pyruvate formate lyase; 11: acetaldehyde dehydrogenase; 12: alcohol dehydrogenase; 13: phosphotransacetylase; 14: acetate kinase.

Lactic acid bacteria can produce diacetyl, acetoin, butanediol and other substances in the process of metabolizing citrate, and the secretion of related substances does not require specific transporters. In citric acid metabolism (Figure 4), extracellular citric acid is transported to the cell through membrane-associated permease, such as 2-hydroxycarboxylate transporter (2-HCT) (Bandell et al., 1997). After citrate enters the cell, it is converted into acetate and oxaloacetate under the catalysis of citric acid lyase complex. Then, oxaloacetate is decarboxylated by oxaloacetate decarboxylase (OAD) to produce pyruvate and carbon dioxide. Subsequently, pyruvate can be metabolized in lactic acid bacteria to produce different end products, including lactate, formate, acetate and ethanol (Sarantinopoulos et al., 2001), as well as important aromatic compounds diacetyl, acetoin and butanediol (Figure 4). Some strains of lactic acid bacteria cannot convert citric acid to pyruvate, but use citrate transporter to generate succinic acid through malic acid and fumaric acid (Torino et al., 2005). Acetaldehyde can be directly catalyzed by pyruvate decarboxylase or pyruvate oxidase, or it can be produced indirectly by the intermediate product acetyl-CoA catalyzed by pyruvate dehydrogenase (Bekal et al., 1998). Diacetyl is produced by the metabolic intermediate α-acetolactate through oxidative decarboxylation (Drinan et al., 1976), and at the same time, α-acetolactate can also be catalyzed by α-acetolactate decarboxylase or through non-oxidative decarboxylation to produce acetoin. When α-acetolactate decarboxylase is inactivated and NADH-oxidase is overexpressed, α-acetolactate can be efficiently converted into diacetyl (Hugenholtz et al., 2000).

In addition, lactic acid bacteria can synthesize sugar alcohols in some processes of sugar metabolism. Sugar alcohols are also called polyols and can be used as thickeners, softeners, stabilizers, etc. Certain lactic acid bacteria (such as *Levilactobacillus brevis* and *Leuconostoc*) can directly reduce fructose to mannitol (Jeske et al., 2018), which can be incorporated into food as a sweetener to exert a variety of beneficial effects. Other hetero-fermentative lactic acid bacteria can synthesize erythritol under certain culture conditions, such as *Oenococcus alcohol, Leuconostoc mesenteroides, and Fructilactobacillus sanfranciscensis* (Ortiz et al., 2013).

#### The Metabolism of Amino Acids Produces Flavor Substances in Fermented Foods

In addition to the above pathways, there are other ways to synthesize acetaldehyde. Deoxynucleic acid aldolase catalyzes the decomposition of thymine to acetaldehyde and glyceraldehyde phosphate. In addition, several amino acids can be converted into the intermediate metabolite pyruvate and finally acetaldehyde, or directly into acetaldehyde (Figure 3). For example, *Streptococcus thermophilus* commonly used in the yogurt industry contains the SHMT gene, which encodes a serine hydroxymethyltransferase (SHMT) with threonine aldolase (TA) activity, which catalyzes the decomposition of threonine into glycine and acetaldehyde (Chaves et al., 2002). In addition, the serine hydroxymethyltransferase encoded by the *glyA* gene can also catalyze the specific reaction of acetaldehyde formation.

In addition, some strains of *Lactiplantibacillus plantarum* can promote the production of aspartic acid related flavor compounds. In *Lacticaseibacillus paracasei*, aspartic acid may be decomposed into diacetyl, acetoin and 1,3-butanediol under the catalysis of aminotransferase (Thage et al., 2005). Some strains of *Lacticaseibacillus casei*, *Lacticaseibacillus rhamnosus*, and *Streptococcus thermophilus* contribute to the production of branched chain amino acid derivatives and aromatic amino acid derivatives (Bancalari et al., 2017). *Streptococcus thermophilus* and *Lacticaseibacillus casei* metabolize to produce 2,3-butanedione and 2,3-pentanedione during milk “laban” fermentation (Chammas et al., 2006).

#### Contribution of Lactic Acid Bacteria in Fermented Food in Synthesizing Flavor Substances

Acetaldehyde, diacetyl, acetoin and butanediol are typical aroma compounds of many fermented dairy products. A variety of lactic acid bacteria play a role in cheese processing. For example, *Lacticaseibacillus paracasei* 4341 can produce aroma and sour substances in Italian long ripened cheeses (Bancalari et al., 2017). *Limosilactobacillus reuteri* INIA P572 enhances the formation of 12 volatile compounds in cheese, but reduces the formation of 5 other volatile compounds (Gómez-Torres et al., 2016). A strain of in Stilton cheese can produce high concentrations of alcohol, organic acids and acetone (Mugampoza et al., 2019). In addition, during the fermentation of cheese, with the participation of *Lacticaseibacillus paracasei* strains, the metabolism of cysteine and methionine will form a sulfuric flavor (Wüthrich et al., 2018). Therefore, the research on the enzymes expressed by lactic acid bacteria will help to screen out suitable strains or combinations of strains to improve or increase the flavor of cheese (Peralta et al., 2016).

In fermented sourdough, lactic acid bacteria also play a role in the formation of flavor substances. For example, during the fermentation of bread sourdough, the combined use of lactic acid bacteria (*Lactiplantibacillus plantarum*, *Lactiplantibacillus plantarum, Furfurilactobacillus rossiae*, and *Lacticaseibacillus casei*) and yeast produces compounds related to the sour aromas of bread, and contributes to the aroma characteristics of bread (Winters et al., 2019). *Lactiplantibacillus plantarum* may promote the production of more C_4_-C_6_ alcohols in sourdough (Liu et al., 2020). In the fermentation of alcoholic beverages, lactic acid bacteria also affect the production of flavor substances. In wine fermentation, lactic acid bacteria can perform malic acid-lactic acid fermentation (MLF), that is, directly produce lactic acid by decarboxylation of L-malic acid. Lactic acid bacteria also affect the production of esters in wine. Some substances have outstanding influence and contribution to fermented products, for example, esters play an important role in the aroma of wine (Cappello et al., 2017). In Bordeaux red wines, lactic acid bacteria are the only bacteria that strongly influence the content of branched hydroxylated esters (ethyl 2-hydroxy-3-methylbutanoate and ethyl 2-hydroxy-4-methylpentanoate) (Gammacurta et al., 2018). The synergistic effect of lactic acid bacteria contributes to the production of flavor substances in vinegar. Some *Lactobacillus* strains, such as *Lentilactobacillus buchneri*, *Limosilactobacillus reuteri*, *Limosilactobacillus fermentum*, and *Levilactobacillus brevis*, may convert 2-acetolactate into acetoin, play important roles in the formation of flavor substances in Zhenjiang aromatic vinegar (Lu et al., 2016).

Based on the above analysis, many lactic acid bacteria will give fermented food a variety of flavor substances. However, due to the variety of lactic acid bacteria, fermentation substrates and flavors produced, it is difficult to fully describe. Therefore, in-depth research on the types of metabolism that produce flavor substances has become critical, which helps to find general strategies for regulating flavor substances. Although the whole genomics, transcriptomics (Liu et al., 2020) and metabonomics (Zhao et al., 2016), and metabolomics are currently developing rapidly, there are still many functional proteins and metabolic pathways that are still unknown. In addition, although the technology for qualitative and quantitative analysis of flavor substances in fermented products has been greatly developed, the effect of the synergy between multiple flavors is still difficult to evaluate. At the same time, since the stability of the fermentation strain is easily affected by many factors, there may still be changes in flavor substances of different product batches. Therefore, the above-mentioned problems constitute the challenges for the in-depth application of the characteristics of lactic acid bacteria to synthesize flavor substances in the food industry in the future.

### Antioxidant Substances

In fermented foods, lactic acid bacteria produce antioxidant substances that are highly safe, and can have a variety of beneficial effects on the human body through food. In recent years, there have been many reports on the synthesis of antioxidant metabolites by lactic acid bacteria. Antioxidant substances have excellent ability to scavenge free radicals and are closely related to human health. Many substances (such as vitamins and polysaccharides) involved in the previous sections have antioxidant capacity, so this section only involves the synthesis of some substances that are mainly used to reflect the antioxidant function of lactic acid bacteria in food. The antioxidant part mainly introduces the metabolism of some phenolic substances by lactic acid bacteria and the limited ability of lactic acid bacteria to synthesize glutathione.

Some lactic acid bacteria (such as *Levilactobacillus brevis*, *Limosilactobacillus fermentum* and *Lactiplantibacillus plantarum*) can metabolize phenolic acid through decarboxylase and reductase (Filannino et al., 2015). Through the decarboxylation and reduction reaction of phenolic acid, hydroxycinnamic acid (*p*-coumaric acid, caffeic acid, ferulic acid and *m*-coumaric acid) and some hydroxybenzoic acid (gallic acid and protocatechuic acid) can be degraded (Rodriguez et al., 2008). For example, after fermentation of blueberries by *Lactiplantibacillus plantarum*, the polyphenols in blueberries can be converted into active phenol metabolites with strong antioxidant and antiproliferative activities (Ryu et al., 2019). The dextransucrase expressed by *Leuconostoc mesenteroides* can convert sucrose into chlorogenic acid glucoside, which increases the water solubility of chlorogenic acid and has better browning resistance (Nam et al., 2017). The fermentation of lactic acid bacteria increases the content of sulforaphane in broccoli puree (Cai et al., 2020). *Lacticaseibacillus rhamnosus*, *Lactobacillus acidophilus* improves the anti-free radical activity of protein extracts from cured meats (Kêska and Stadnik, 2018). *Lactiplantibacillus plantarum* significantly reduces the protein carbonyl content and protein surface hydrophobicity in fermented sausages, and increases the content of total sulfhydryl contents (Ge et al., 2019).

As a natural antioxidant, glutathione is a tripeptide composed of glutamic acid, cysteine and glycine (Wu et al., 2004). Two enzymes are important enzymes involved in glutathione metabolism. Glutathione peroxidase catalyzes the conversion of reduced glutathione to its oxidized form, and glutathione reductase regenerates reduced glutathione (Qiao et al., 2018). Glutathione is rare in Gram-positive bacteria, but it can also be produced in some lactic acid bacteria, such as *Streptococcus thermophilus*, *Enterococcus faecalis*, *Lacticaseibacillus casei*, *Lacticaseibacillus rhamnosus*, *Lactiplantibacillus plantarum*, *Latilactobacillus sakei* and *Ligilactobacillus ruminis*, etc. (Pophaly et al., 2012). Although *Limosilactobacillus fermentum* CECT 5716 cannot synthesize glutathione, it can convert oxidized glutathione taken from the environment into reduced glutathione. This also promotes the release of glutathione to the environment (Surya et al., 2018). As an additive, glutathione makes outstanding contributions to increase dough rheology (Loponen et al., 2008) and promote the hydrolysis of egg white protein (Loponen et al., 2008). For example, the glutathione reductase of *Fructilactobacillus sanfranciscensis* affects the volume of bread by affecting the thiol content in wheat dough and the polymerization and aggregation of gluten (Xu et al., 2018). In addition, glutathione also improves the acid resistance of lactic acid bacteria (Xu et al., 2018).

Numerous studies have shown that antioxidants, as a class of metabolites of great concern, have also played an important supplementary role in explaining the mechanism of lactic acid bacteria as probiotics. For example, *Lactiplantibacillus plantarum* Y44 plays an antioxidant role by free radical scavenging ability and activating the Nrf2 signaling pathway. In injury-induced Caco-2 cells, it can down-regulate the expression of inflammatory-related cytokines IL-8 and tumor necrosis factor-α, and at the same time enhance the expression of intracellular tight junction proteins β-catenin and E-cadherin (Mu et al., 2019). Therefore, in-depth study of its metabolic pathways has an important guiding role for the promotion of lactic acid bacteria in human health, or the discovery of more efficient antioxidant substances used in food.

## Metabolic Engineering Strategies Application in Lab

Metabolic engineering refers to the use of synthetic biology and systems biology methods to improve the utilization of existing or create new biosynthetic metabolic pathways. In the food industry, the main purpose of applying metabolic engineering strategies in lactic acid bacteria is to increase the production of certain metabolites of lactic acid bacteria (such as exopolysaccharides, sugar alcohol compounds, vitamins and bacteriostatic peptides, etc.).

For example, using central carbon metabolism pathways (including glycolysis, gluconeogenesis, pentose pathway and citric acid cycle, etc.) as the target, the output of a certain target product can be increased by changing the metabolic flux. The research methods mainly include expanding existing metabolic pathways and constructing novel metabolic pathways. (1) Expanding existing pathways means overexpression of rate-limiting enzyme genes in related biosynthetic pathways on the basis of existing metabolic pathways, and inhibiting competitive metabolism. For example, pyruvate is a key substance in the central carbon metabolism pathway, and its content is very important for the synthesis of organic acids such as lactic acid, α-acetolactate, acetic acid, and formic acid. Therefore, in the production of lactic acid, methods such as adjusting the metabolic flow of pyruvate and enhancing the expression of lactate dehydrogenase can be used to achieve high yields. In addition, the use of metabolic engineering can also effectively optimize the ability of lactic acid bacteria to degrade proteins, which in turn helps to control protein hydrolysis and help obtain the most optimized food processing technology. (2) The construction of a novel metabolic pathway refers to the expression of multiple genes encoding a pathway-specific enzyme in a non-natural host to construct a new pathway. This is generally due to the fact that although all possible genetic manipulation procedures and metabolic engineering strategies are used in natural strains, sometimes the production titer of the required metabolites is still very low or does not meet the requirements. For example, by integrating the gene of the short-chain dehydrogenase encoded by *Corynebacterium glutamicum* into *Pediococcus acidilactici*, the resulting genetically engineered strain synthesizes higher yield of D-lactic acid (Qiu et al., 2020).

In short, with the development of the CRISPR-Cas9 system and other gene editing systems (Hidalgo-Cantabrana et al., 2019), a variety of metabolic engineering strategies can be used to meet the growing demand for microbial metabolites in industrial production.

## Conclusion and Perspectives

Although the lactic acid bacteria had been used in fermentation products for a long time. The lactic acid bacteria work in a vast array of ways that its functional characteristics are quite different. In the food industry, a variety of bacteria’s can ferment a variety of substrates into products or produce a variety of industrial raw materials during the fermentation process. Due to the limited output of its products, despite some of the products being valuable, some lactic acid bacteria may not be enough to be used as industrial-grade bacteria. Therefore, the use of synthetic biology technology to carry out targeted transformation of strains can help synthesize certain products in high yield. The constructed lactic acid bacteria engineering strain can be used to increase the production of organic acids such as γ-aminobutyric acid and lactic acid, exopolysaccharides, vitamins and other products, and can also be used to express enzymes that decompose proteins and polysaccharides. Among them, *Levilactobacillus brevis*, *Lactococcus lactis*, *Lactiplantibacillus plantarum* and other lactic acid strains have carried out excellent metabolic engineering work. In addition, the use of industrial wastes such as whey as production substrates is also an important way to alleviate the current situation of resource shortages. However, in some cases, when the inherent flora structure in the human intestine encounters excessive intake of certain lactic acid bacteria, it may cause the original flora structure to become unbalanced and cause human discomfort. Therefore, the understanding of strain metabolism can avoid some damage to human health.

In addition, the development of modern biotechnology such as multi-omics technology and gene editing technology and its application in lactic acid bacteria have also strongly promoted in-depth research on the metabolism of lactic acid bacteria. The research on the metabolic pathways and metabolic characteristics of lactic acid bacteria will help further guide the application of lactic acid bacteria in the food industry and human health industry.

## Author Contributions

All authors listed have made a substantial, direct and intellectual contribution to the work.

## Conflict of Interest

The authors declare that the research was conducted in the absence of any commercial or financial relationships that could be construed as a potential conflict of interest.
